# Editorial: Responsible Research and Innovation as a toolkit: indicators, application, and context

**DOI:** 10.3389/frma.2023.1267951

**Published:** 2023-08-28

**Authors:** Tobias Buchmann, Marion Dreyer, Matthias Müller, Andreas Pyka

**Affiliations:** ^1^Department of System Analysis, Zentrum für Sonnenenergie- und Wasserstoff-Forschung Baden-Württemberg, Stuttgart, Baden-Württemberg, Germany; ^2^DIALOGIK gemeinnuetzige Gesellschaft für Kommunikations- und Kooperationsforschung mbH, Stuttgart, Baden-Württemberg, Germany; ^3^Department of Innovation Economics (520i), University of Hohenheim, Stuttgart, Germany; ^4^Department of Economics, University of Insubria, Varese, Italy; ^5^Flying Faculty, Department of Economics, Turkish-German University, Istanbul, Türkiye

**Keywords:** Responsible Research and Innovation (RRI), innovation, innovation management, toolkit approach, responsible research

Responsible Research and Innovation (RRI) is a concept aimed at ensuring the acceptability, desirability and sustainability of research and innovation activities. It encompasses a range of principles and processes to promote ethical decision-making and societal engagement in science, technology and innovation (STI; Stilgoe et al., 2013; von Schomberg, 2013). While RRI has gained traction in various contexts, there are open challenges in translating the concept into practical action and ensuring its long-term impact. The Research Topic, which this editorial introduces, explores the role of toolkits in fostering RRI practices and their potential for implementing responsible innovation practices. RRI is not a fixed concept but rather a collection of principles, methods and approaches that can be adapted and applied to specific contexts (Yaghmaei and Poel, 2020; Kwee et al., 2021). Thus, the articles related to the topic highlight the importance of recognizing the diverse purposes and objectives of RRI within different fields of application.

One point the Research Topic makes is that toolkits can facilitate the implementation of RRI. However, it makes also clear, that there is a risk of excessive standardization. If applied excessively, they can overshadow the broader rationales and purposes of RRI. This can lead to the loss of core principles and a narrow focus on methods rather than the underlying objectives of RRI. Furthermore, RRI is not a static concept but rather an ongoing process of reflection and adaptation. It requires researchers and innovators to continually reassess their actions and consider the broader social and environmental implications of their work. Contextual adaptation of tools is crucial in this regard, as it acknowledges the unique characteristics and challenges of different fields of application such as technological domains and stakeholders.

More fundamentally, the Research Topic highlights that there is increasing need to integrate responsibility into innovation processes. Innovation should not be driven primarily by technological and economic considerations and give due consideration to ethical and social dimensions. With the recognition of sustainability as a global challenge, responsibility should be embedded at the core of innovation activities. Thereby, innovators can ensure that their solutions contribute to a more sustainable future. In this respect, toolkits play a crucial role in supporting the implementation and dissemination of RRI principles. The following articles provide guidance and practical tools for researchers and organizations to navigate the complexities of responsible innovation.

A first article on “*Translating tools and indicators in territorial RRI*” by Völker et al. sheds light on the challenges associated with the multiple territorial translations of RRI. It emphasizes the significance of building upon pre-existing relationships and collaborations, highlighting that RRI should not start from scratch but rather continue ongoing work with pre-set objectives and aims. The article warns against the over-standardization of RRI through “toolification” and the use of quantitative indicators. Instead, it advocates for evaluative inquiry methods to monitor RRI performance. The article also highlights the need to ensure a lasting legacy for RRI projects and initiatives. It explores the concept of toolkits as mechanisms for preserving and disseminating RRI practices beyond the duration of individual projects. By developing an RRI toolkit, project outcomes and insights can be integrated into future research infrastructures and initiatives.

Article 2 on “*Addressing responsibility in innovation processes for sustainability: lessons for responsible management of sustainable innovation form a systematic literature review*” by Mangelkramer underscores the need to analyze the impact of a sustainability agenda on research and innovation processes for system transition. It acknowledges two critical gaps in the existing literature: the lack of insights into the implications for managing innovation processes at the organizational level and the limited integration of responsibility in Sustainable Innovation (SI). The article argues that without strategically embedding responsibility, there is a risk of creating partially sustainable and irresponsible socio-technical system changes through business innovation activities. To bridge this gap, the article proposes an extended innovation process model for sustainability that places responsibility at the core of innovation activities. Drawing from the framework of RRI, responsibility becomes a guiding principle, steering innovation toward sustainable and socially responsible outcomes.

Article 3 on “*Building a responsible innovation toolkit as project legacy*” by Stahl and Bitsch delves into the use of toolkits as mechanisms for ensuring the long-lasting impact and legacy of RRI projects. The article acknowledges the challenge of securing a lasting impact, as traditional mechanisms such as follow-on project funding or market-oriented venues are not readily available for RRI. To address this challenge, the article explores the development of an RRI toolkit within the EU-funded Human Brain Project (HBP). The toolkit is designed to support activities in the EBRAINS research infrastructure, which serves as the main legacy mechanism of the HBP. By providing resources and guidance, the toolkit aims to extend the influence of RRI beyond the project's funding period.

Article 4 on “*Responsibly shaping technology innovation for the energy transition: an RRI indicator system as a tool*” by Buchmann et al. provides insights on using RRI as a tool for steering energy transition innovations toward social acceptance. RRI shapes innovation processes, addressing complexity and uncertainty. It can help to guide the energy transition toward societally beneficial outcomes also beyond clean energy and carbon neutrality, considering also other grand societal challenges such as biodiversity and social justice in close interaction with societal actors. RRI allows for the design of systems and processes that increase the probability of socially desirable and accepted innovations. Toolkits facilitate the practical application of RRI by providing tangible indicators and guidelines. The article proposes an RRI base indicator system for energy transition innovations. It is designed to promote early integration of environmental and social aspects, facilitate responsible team formation, and enable progress monitoring across relevant RRI dimensions.

The four articles shed light on the essential aspects of RRI. They highlight the importance of integrating responsibility into innovation processes, the need for contextual adaptation of RRI and the use of toolkits to ensure a lasting impact. RRI goes beyond the notion of mere compliance, it fosters proactivity, ethical deliberation and societal engagement. It calls for researchers, policymakers and stakeholders to collaborate in shaping the direction of scientific and technological progress, considering the wider implications and consequences of their work.

## Author contributions

TB: Writing—original draft, Writing—review and editing. MD: Writing—original draft, Writing—review and editing. MM: Writing—original draft, Writing—review and editing. AP: Writing—original draft, Writing—review and editing.
